# A chameleon-like core–shell organic/lanthanide flexible crystal waveguide for bandwidth and colour tunability

**DOI:** 10.1039/d6sc01977c

**Published:** 2026-05-29

**Authors:** Melchi Chosenyah, Mehdi Rohullah, Avulu Vinod Kumar, K. V. Jovan Jose, Rajadurai Chandrasekar

**Affiliations:** a School of Chemistry, Centre for Nanotechnology, University of Hyderabad Prof. C. R. Rao Road, Gachibowli Hyderabad 500046 Telangana India r.chandrasekar@uohyd.ac.in

## Abstract

Optical fibers capable of dynamically generating and/or transporting narrow/broadband spectral signals in the visible spectral region based on the input light, much like how a chameleon changes its color, are quintessential for developing visible light communication devices. Here, we demonstrate a mechanically flexible, blue-violet fluorescent 2-(4,4′-bis(2,6-di(1*H*-pyrazol-1-yl)pyridin-4-yl)biphenyl) (BPP) crystal waveguide surface coordinated to red fluorescent Eu(tta)_3_. The BPP microcrystal waveguide, acting as the core, with BPP-Eu(iii) as the shell, provides a hybrid platform for broad and narrow band signal transmission. Depending on the input light and the absorption of the core or shell, the crystal acts as an active–active, passive–active, or passive–passive light-generating and/or transporting optical waveguide. Notably, the pseudo-plasticity of the core–shell hybrid waveguide enables modulation of the signal output direction without compromising its optical performances. The development of such smart optical waveguides has enormous potential for visible light communication and selective light-based microprecision sensing applications.

## Abstract 2

A blue-emissive microcrystal core coordinated with a red-emissive Eu(iii) shell optical waveguide enable dynamically transduce light *via* active–active, passive–active, and passive–passive modes governed by the excitation and absorption wavelengths.

## Graphical abstract

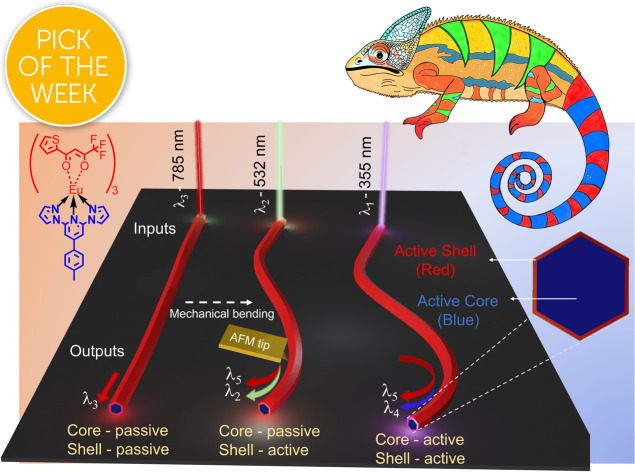

Chameleon-like crystalline waveguides that can selectively generate and transport light across the blue-to-NIR spectrum with tunable bandwidth hold significant promise for advancing visible light communication (VLC) technologies.^1,2^ Unlike silicon or silicon-based complementary metal oxide semiconductor materials, organic waveguides can function as active and passive optical waveguides depending on whether the input optical signal is within or outside the molecule's absorption window.^3–17^ We have previously demonstrated that the microcrystals of 2-(4,4′-bis(2,6-di(1*H*-pyrazol-1-yl)pyridin-4-yl)biphenyl) (BPP) serve as both passive and active waveguides while exhibiting mechanical compliance.^17,18^ Active^4,5,7–16^ and passive^3,5,6,16^ organic waveguides, when combined with energy transfer and reabsorption properties, can be utilized to create various optical components such as directional couplers,^5,19^ add-drop filters,^20^ wavelength division multiplexers,^21,22^ and photonic circuits.^5,18–25^

Numerous one-dimensional (1D) organic crystalline waveguides capable of transporting visible light have been demonstrated.^26–33^ Additionally, several core–shell strategies for organic optical waveguides have been reported.^15,34–42^ However, spontaneous generation and propagation of narrow and/or broadband optical signals involving active/passive transduction and sensitized energy transfer in a single organic waveguide remains challenging. Typically, organic crystals exhibit broadband emission, while lanthanides display narrow band *f–f* transitions. Recently, X.-D. Wang *et al.* reported a core–shell strategy to coat the rigid nanocrystal surface with suitable organic acceptor or donor molecules, thereby modifying the broadband active emission properties of the crystals.^9,12,15^ Nevertheless, developing a chameleon-like optical waveguide capable of generating and transporting (i) dynamic colours across the visible spectral regions, (ii) either narrowband or broadband signals, and (iii) a combination of narrow and broad bands presents a significant challenge. This task necessitates the layering of organic and lanthanide optical materials. A viable chemical approach to achieve this desired goal involves selecting a mechanically flexible, microcrystal derived from an organic fluorescent (FL) ligand. This microcrystal can then be coordinatively reacted at the surface with an appropriate lanthanide metal to form an organic–lanthanide core–shell hybrid optical waveguide (Fig. 1).

**Fig. 1:**
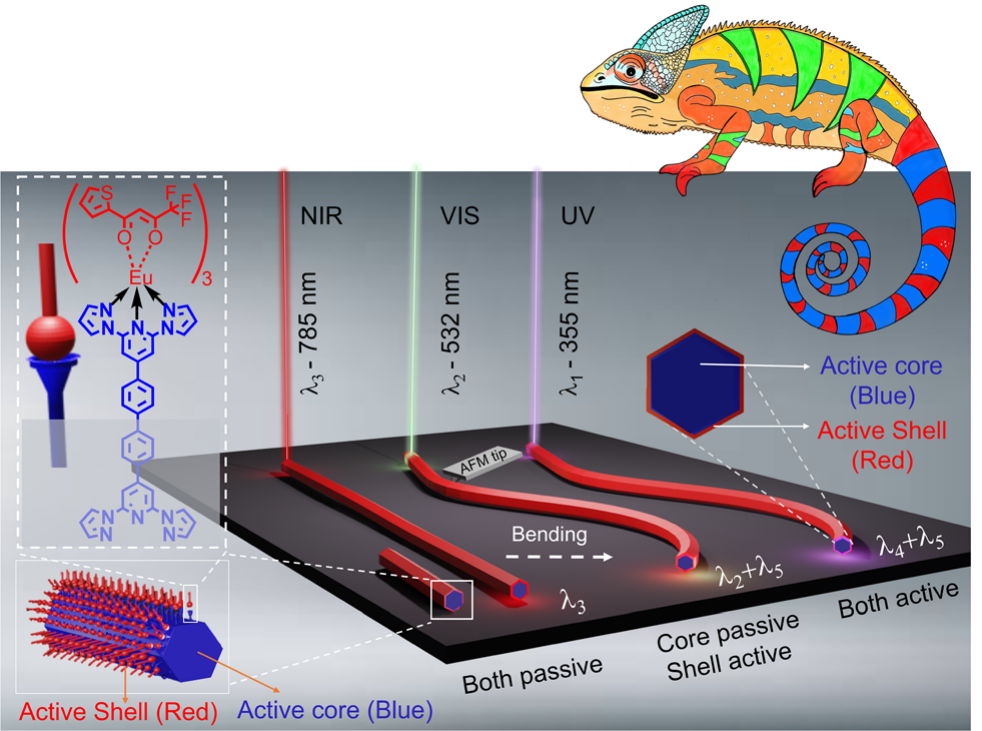
Illustration of a core–shell organic/lanthanide hybrid crystal waveguide showing active/passive input-dependent optical waveguiding.

Apart from BPP crystal's active/passive waveguiding propensity^17,18^ and mechanical flexibility,^18^ its unexplored chemical feature is the available tridentate 2,6-bispyrazolylpyridine unit. For example, Ln(tta)_3_ hydrates (tta: thenoyltrifluoroacetonate) can react with BPP ligands to produce a nine-coordinated narrowband luminescent lanthanide complex.^43–47^ Here, we demonstrate a “coordination chemistry at the crystal surface” strategy^42^ to fabricate organic/lanthanide core–shell hybrid optical waveguides from blue-violet FL BPP microcrystals. The exposed tridentate sites at the BPP microcrystal surface selectively and coordinatively react with red-FL Eu(tta)_3_ hydrate. The obtained core–shell hybrid optical waveguide selectively transduces broadband blue-violet emission from the organic ligand (the unreacted core), narrowband red emission from the Eu(iii) in the shell, or a purple colour resulting from the mixing of blue and red colours, depending upon the optical absorbance of the input light (Fig. S1). For a UV laser input, the waveguide's core and shell actively produce and guide the broad blue-violet band and narrow red band signals, respectively. On the other hand, the waveguide core and shell passively and actively guide narrow laser and red signals, respectively, for the 532 nm input. For a 785 nm input, both the core and shell passively guide the laser light. Furthermore, the mechanical flexibility of BPP crystals provides additional prospects for manipulating optical signals, thus illustrating a futuristic, flexible, organic/lanthanide hybrid crystal optical waveguide technology delivering active/passive tunable light output. This chameleon-like behaviour to dynamically adjust the signal output for different inputs makes these waveguides versatile for sensing and circuit applications.

The BPP molecule was synthesized using the reported procedure.^46^ The microrods of BPP were obtained by slow evaporation of 50 µL BPP solution (*c* ≈ 0.35 × 10^−3^ M; 0.2 mg mL^−1^ in dichloromethane (DCM)) under hexane atmosphere at room temperature (Fig. 2a).^18^ The self-assembled one-dimensional microstructures exhibited hexagonal and rectangular cross-sections, and their characteristics were examined in field emission scanning electron microscopy (FESEM) (Fig. S1 and S2). The tubular morphology observed in certain crystals results from solvent etching or the slow growth of high-energy crystal facets.^18^ The exposure of BPP ligands on the surface of the hexagonal microcrystals was investigated by analyzing the planes of the macrocrystal.^18^ This analysis revealed that the (110), (−210), and (−120) planes of the hexagonal microcrystals were exposed (Fig. 2b and c). From these identified planes, it is evident that the tridentate ligands are presented on the surface of the BPP's hexagonal microcrystals (Fig. 2d).

**Fig. 2:**
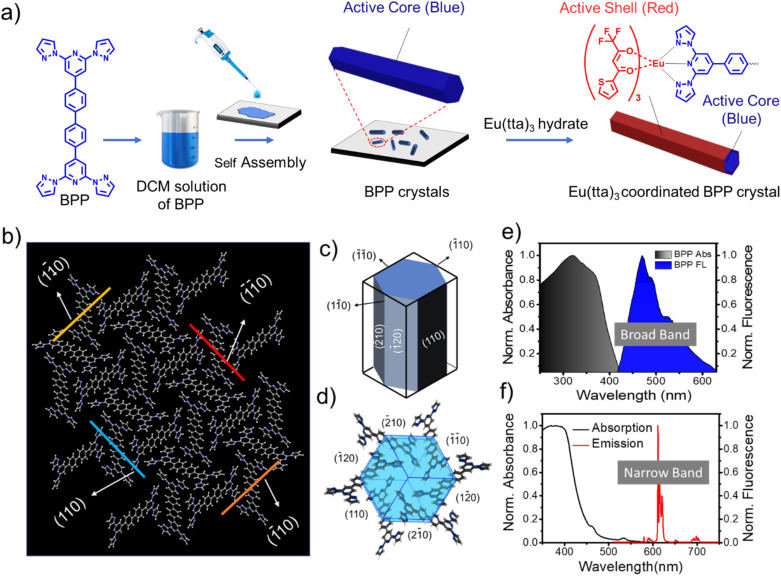
(a) Schematic elucidation of the formation of core–shell organic/lanthanide hybrid hexagonal microcrystals in DCM *via* supramolecular self-assembly. (b) Single-crystal X-ray structure depicting the molecular packing of a rectangular-shaped BPP crystal with the corresponding facets. (c) Facets of hexagonal BPP microcrystal compared with a macrocrystal. (d) Growth morphology of the BPP crystal. (e) Solid-state absorption and emission spectra of the BPP ligand. (f) Solid-state absorption and emission spectra of Eu(tta)_3_ hydrate.

The crystalline microrod's solid-state optical absorption spans from the UV region to 425 nm, with a maximum absorption at 320 nm. BPP showed blue-violet FL in the solid state, which extends from ≈400 to 650 nm with *λ*_max_ at 470/490/526 nm (Fig. 2e). The optical absorption of Eu(tta)_3_ hydrate falls in the UV region and extends up to 600 nm, whereas it emits sensitized red FL (580–700 nm) from the hypersensitive ^5^D_0_-^7^F_*J*_ (*J* = 0–4) transitions of the Eu(iii) ion (Fig. 1f and S3a). Interestingly, the emission of BPP and absorption of Eu(tta)_3_ overlap, facilitating radiative energy transfer from the ligand to the metal ion. This energy transfer enables enhancement of the red emission intensity from Eu(iii) (Fig. S3b and c).

To test the coordination chemistry-assisted surface coating of Eu(tta)_3_ on the BPP crystal surface, a hexane solution (20 µL) of Eu(tta)_3_ hydrate (*c* ≈ 0.115 × 10^−3^ M) was dropped onto the BPP crystals. Here, the exposed tridentate ligand available at the crystal surface drives the water molecules out of Eu(tta)_3_ hydrate, forming a thin layer of 2,6-bispyrazolylpyridine-Eu(tta)_3_ coordination complex on the BPP crystal surface. The FESEM image established crystal structural integrity even after Eu(iii) complex formation at the crystal surface (Fig. S4a and b). Optical waveguiding studies further validated the presence of BPP-Eu(tta)_3_ complexation (Fig. S4c–f). The energy dispersive X-ray analysis (EDX) confirmed the Eu content (Fig. S4g). Selected area electron diffraction (SAED) obtained *via* transmission electron microscopy (TEM) showed distinctive diffraction patterns for BPP and Eu-coordinated heterostructures (Fig. S5). Additionally, the Raman spectra of the hexagonal rods of the BPP ligand, those reacted with the nine-coordinated Eu(III) complex and pure Eu(tta)_3_ hydrate, have demonstrated new peaks and peak shifts that support the presence of a layer of the Eu complex on the surface (Fig. 3a–c). Furthermore, because of the formation of a thin inorganic layer around the BPP microcrystal, the signal intensities of BPP dominate over those originating from the thin layer of the Eu complex. Increasing the concentration of Eu(tta)_3_ hydrate will in turn enhance the Eu-centered emission intensity (Fig. S6). The far-IR region of the spectra exhibited a new asymmetric peak with a base width of about 298–365 cm^−1^. The peak asymmetry is due to the contribution of two types of *ν*_(Eu–N)_ in the experimental spectrum. Moreover, the calculated Raman spectrum also showed a new peak for *ν*_(Eu–N)_ = 342 cm^−1^, confirming the coordination of BPP with Eu(tta)_3_ (Fig. 3c). The 1603 cm^−1^ peak in BPP and the 1086 cm^−1^ peak in Eu(tta)_3_ hydrate are observed in both the experimental and calculated spectra with different intensities. The 246 cm^−1^ peak of BPP and the 260 cm^−1^ peak (*ν*_(Eu–O)_) of Eu(tta)_3_ hydrate merged in the experimental spectrum, giving rise to an asymmetric peak. These results confirmed the formation of the BPP-Eu(tta)_3_ coordination complex on the surface of BPP microcrystals.

**Fig. 3:**
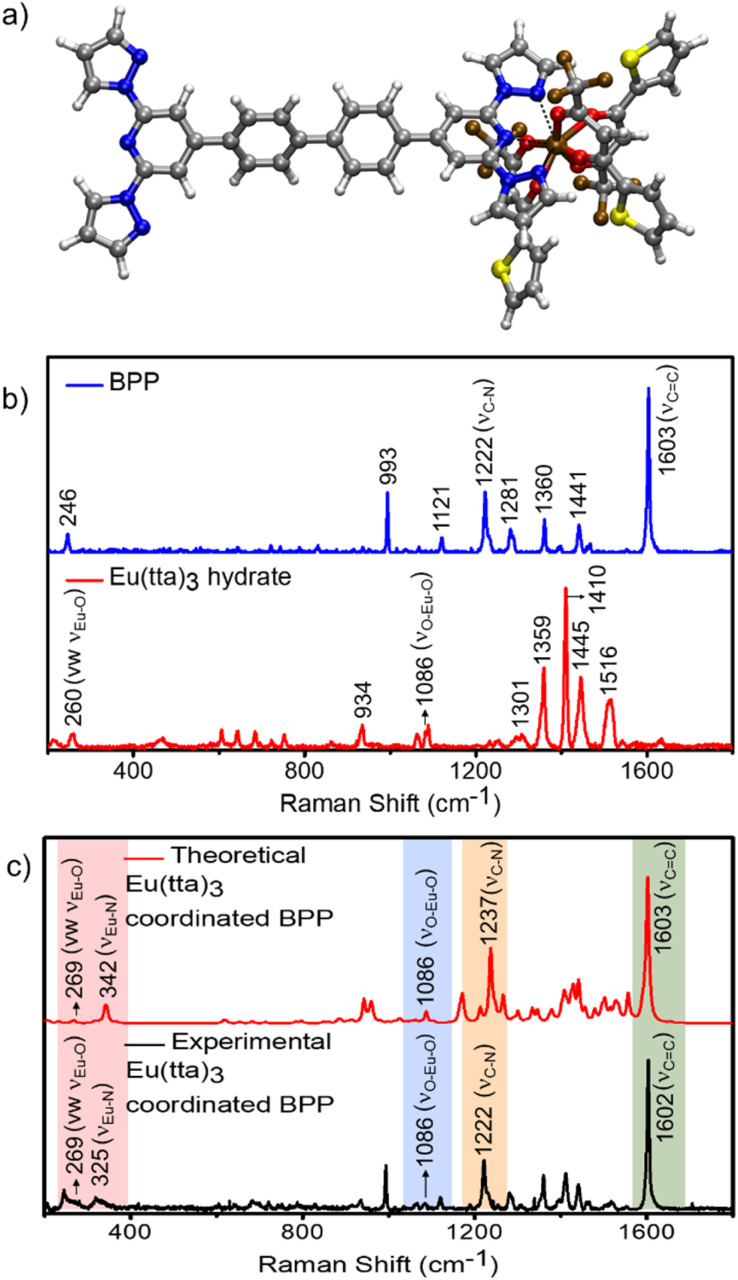
(a) Energy optimized structure of Eu(tta)_3_ coordinated to BPP. (b) Micro Raman spectra of BPP and Eu(tta)_3_ hydrate. (c) Theoretical (B3LYP level of theory with the Def2svp basis set, using the Gaussian09 suite of programs) and experimental Raman spectra of Eu(tta)_3_ coordinated BPP. Excitation wavelength *λ*_ex_ = 785 nm.

To understand the mechanophotonic properties of the hybrid microstructure, a BPP crystal (*L* ≈ 286 µm) was selected, and the optical waveguiding studies were performed (Fig. 4a and b). A 355 nm continuous-wave (CW) laser was focused onto one end of the microcrystal from the bottom to provide excitation. The produced blue-violet FL was collected from the opposite end of the microcrystal. The excitation position was systematically varied along the length of the microcrystal while maintaining a fixed collection position at one end. The corresponding spectra were plotted in Fig. 4c. The optical loss coefficient, *α*′ (a parameter to estimate the waveguiding efficiency) of the optical waveguide was calculated to be *α*′ = 0.0733 dB µm^−1^ by fitting the plot of *I*_tip_/*I*_body_*vs.* distance between excitation and collection positions (Fig. 4k). The same microcrystal's surface was treated with Eu(tta)_3_ hydrate to form a nine-coordinated Eu(iii)-BPP complex as a thin layer (Fig. 4d–f). The FL spectrum recorded at the laser irradiation point/opposite terminal revealed two bands corresponding to the BPP ligand at 450 nm (broadband), as well as the Eu complex in the region around 630 nm (narrowband) (Fig. 4f and S4d). Thereby, confirming the presence of the Eu complex (shell) on top of the microcrystal (core) due to the formation of a hybrid organic/lanthanide heterostructure. The optical waveguiding studies performed on the core–shell crystal confirmed the dual signal propagation (*λ*_max_ of 450 nm from BPP and 630 nm from Eu) to the terminals (Fig. 4f) with a *α*′ of 0.2009 dB µm^−1^ (Fig. 4k).

**Fig. 4:**
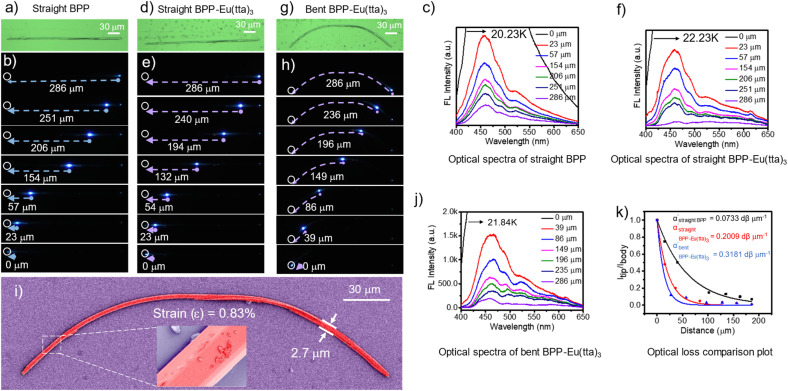
Confocal optical image and FL images of BPP microcrystals in (a and b) uncoordinated, (d and e) Eu(tta)_3_ coordinated (straight), and (g and h) Eu(tta)_3_ coordinated (bent) configurations. The excitation position-dependent FL spectra obtained in the (c) uncoordinated BPP, (f) straight Eu(tta)_3_ coordinated BPP, and (j) bent Eu(tta)_3_ coordinated BPP microcrystals. (i) Color-coded FESEM micrograph of a BPP microcrystal coordinated with Eu(tta)_3_ hydrate. Inset: magnified image of the hexagonal morphology. (k) The plot of *I*_tip_/*I*_body_*vs.* distance used for the optical loss coefficient calculation for uncoordinated, coordinated straight, and coordinated bent BPP microcrystals.

Later, a confocal microscope attached to an atomic force microscope (AFM) cantilever tip was used to investigate the micromechanical compliance of the hybrid heterostructure waveguide. For that, the left terminal of the hybrid waveguide was gently pushed with the cantilever tip by applying a force in the forward direction, which confirmed the crystal's flexibility. Later, a similar force was applied to the right terminal by implementing the same procedure, resulting in a bow-shaped curved hybrid waveguide (Fig. 4g–i). This mechanical bending imparted a strain of 0.83% on the hybrid waveguide (Fig. S7). The FESEM image of the hybrid waveguide revealed a thickness of 2.7 µm (Fig. 4i). Despite the strain due to mechanical bending, the hybrid waveguide obtained from an elastically flexible BPP microcrystal did not revert to its original shape after applied stress removal because of its pseudo-plasticity^19^ (owing to the crystal surface and substrate adhesive interaction, the elastic microcrystal behaves akin to plastic). The strained hybrid waveguide was subjected to a 355 nm laser to investigate its optical waveguiding characteristics. As expected, the strained hybrid waveguide showed a slightly higher optical loss of 0.3181 dB µm^−1^, attributed to the crystal bending and scattering loss (Fig. 4k).

To showcase the intended generation of multiple spectral colours, bandwidths, and transportability within the core–shell structure, a 238 µm-long hybrid crystal waveguide was specifically chosen (Fig. 5a, inset of Fig. 5d). Illuminating the left end of the hybrid crystal waveguide with a 355 nm laser resulted in FL due to optical absorption from both BPP and Eu(iii) and their propagation to the opposite end of the waveguide (Fig. 5a and d). Here, both the core and shell act as active waveguides. Conversely, when the same waveguide was excited with a 532 nm laser at one end, only the red FL from the Eu complex was actively transduced to the other end, given that its absorption region aligned well with the excitation source (Fig. 5b and e). In contrast, the absorption of BPP molecules ends at 440 nm, enabling passive guidance of the 532 nm green light (Fig. S3c and S8). In addition to the red FL from Eu, BPP's Raman signals were observed. This led to the creation of a dual-mode passive–active light transduction through the core and shell, respectively, within the hybrid crystal waveguide.

**Fig. 5:**
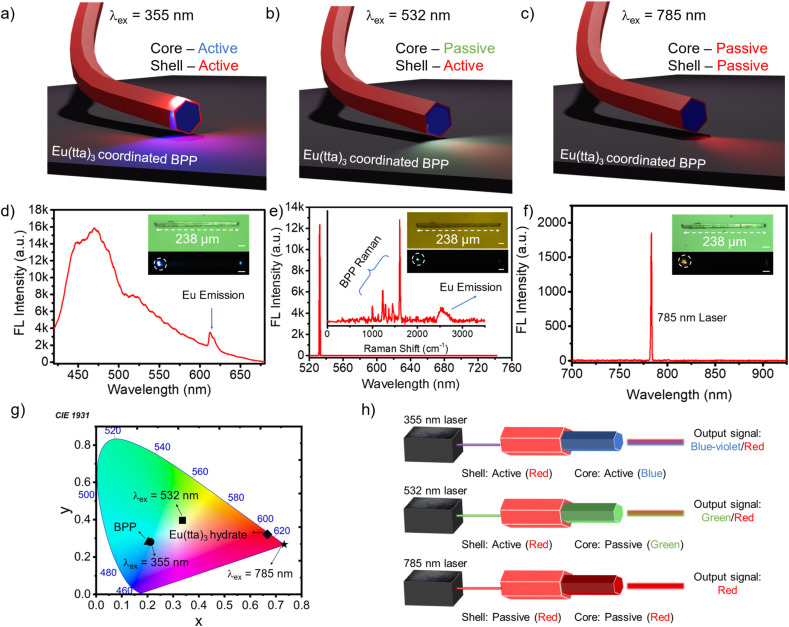
Graphical representation of the dynamic performance of the Eu(tta)_3_ coordinated BPP hybrid crystal waveguide under excitation with (a) 355 nm, (b) 532 nm, and (c) 785 nm lasers. FL spectrum collected at the left tip, when excited the crystal with (d) 355 nm, (e) 532 nm, and (f) 785 nm lasers (inset: confocal and FL images of the crystal under excitation with 355, 532 and 785 nm lasers, respectively). Scale bar: 20 µm. (g) The CIE 1931 diagram depicts the colour coordinates for BPP, Eu(tta)_3_ hydrate, and Eu(tta)_3_ coordinated BPP when irradiated with different wavelength lasers. (h) Graphical representation of the light transduction mechanisms and output colours for different lasers.

Furthermore, experimentation involved the excitation of the same hybrid crystal waveguide with a 785 nm laser, situated away from the absorption bands of both BPP and Eu(iii), at one of its terminals (Fig. 5c and f). Consequently, the same laser light passively propagated to the other end of the hybrid microcrystal, confirming the passive-only light transportability of the core and shell of the hybrid crystal waveguide (Fig. 5c and f). The graphical representation in Fig. 5h summarizes the optical performance of the constructed hybrid crystal waveguide, outlining the signal outcome based on the input laser source. The CIE diagram visually illustrates the emission changes for each recorded output (Fig. 5g, h and Table S1).

## Conclusions

In conclusion, we have successfully demonstrated the optical waveguiding capabilities of highly flexible hybrid organic/lanthanide core–shell crystals, exhibiting chameleon-like dynamic colour-changing properties. This work not only showcases advancements in flexible organic–inorganic crystal optical waveguides but also emphasizes the untapped potential of crystals with metal ligation features. The crystals of the BPP-Eu(tta)_3_ hybrid structure successfully guided blue-violet and red active–active signals with a 355 nm laser, green and red passive–active signals with a 532 nm laser, and red passive signals with a 785 nm laser, with low optical loss, demonstrating their versatile input-dependent waveguiding nature. Such hybrid materials composed of organic/inorganic wavelength-tunable active/passive flexible hybrid crystal waveguides are exemplary materials for advancing organic photonic technologies. Such exemplary photonic structures capable of transducing combined broadband and narrowband optical signals are necessary for VLC technologies.

## Author contributions

The manuscript was written with contributions from all authors. All authors have approved the final version of the manuscript.

## Conflicts of interest

There are no conflicts to declare.

## Supplementary Material

SC-OLF-D6SC01977C-s001

## Data Availability

The supporting data have been provided as part of the supplementary information (SI). Supplementary information: synthesis, experimental methods, characterisation and computational details. See DOI: https://doi.org/10.1039/d6sc01977c.

## References

[cit1] Ismail S. N., Salih M. H. (2020). AIP Conf. Proc..

[cit2] Khapre A., Hazarika J., Chandrasekar R. (2025). Nat. Commun..

[cit3] Su Y., Zhang Y., Qiu C., Guo X., Sun L. (2020). Adv. Mater. Technol..

[cit4] Zhao Y. S. , Organic Nanophotonics: Fundamentals and Applications, Springer, Berlin, 2014

[cit5] Chandrasekar R. , Mechanophotonics for Organic Photonic Integrated Circuits, IOP Publishing, London, 2024

[cit6] Basak S., Chandrasekar R. (2013). J. Mater. Chem. C.

[cit7] Takazawa K., Kitahama Y., Kimura Y., Kido G. (2005). Nano Lett..

[cit8] Hu F., Zhang G., Zhan C., Zhang W., Yan Y., Zhao Y., Fu H., Zhang D. (2014). Small.

[cit9] Zhang J.-X., Zhao S., Lu Z., Wang X.-D. (2025). Adv. Funct. Mater..

[cit10] Matsuo T., Kuwabara J., Kanbara T., Hayashi S. (2023). J. Phys. Chem. Lett..

[cit11] Zhuo M., Tao Y., Wang X., Wu Y., Chen S., Liao L., Jiang L. (2018). Angew. Chem., Int. Ed..

[cit12] Chen S., Zhuo M.-P., Wang X.-D., Wei G.-Q., Liao L.-S. (2021). PhotoniX.

[cit13] Zhuo M.-P., Fei X.-Y., Tao Y.-C., Fan J., Wang X.-D., Xie W.-F., Liao L.-S. (2019). ACS Appl. Mater. Interfaces.

[cit14] Li Z.-Z., Wu J.-J., Wang X.-D., Wang K.-L., Zhang S., Xie W.-F., Liao L.-S. (2019). Adv. Opt. Mater..

[cit15] Jiang J.-H., Zhao S., Zhang J.-X., Lv Z.-J., Song J., Sun Y., Liao L.-S., Wang X.-D. (2024). Nano Lett..

[cit16] Annadhasan M., Basak S., Chandrasekhar N., Chandrasekar R. (2020). Adv. Opt. Mater..

[cit17] Chandrasekhar N., Mohiddon M. A., Chandrasekar R. (2012). Adv. Opt. Mater..

[cit18] Kumar A. V., Rohullah M., Chosenyah M., Ravi J., Venkataramudu U., Chandrasekar R. (2023). Angew. Chem., Int. Ed..

[cit19] Annadhasan M., Agrawal A. R., Bhunia S., Pradeep V. V., Zade S. S., Reddy C. M., Chandrasekar R. (2020). Angew. Chem., Int. Ed..

[cit20] Kumar A. V., Mamonov E., Murzina T., Chandrasekar R. (2023). Adv. Opt. Mater..

[cit21] Kumar A. V., Godumala M., Ravi J., Chandrasekar R. (2022). Angew. Chem., Int. Ed..

[cit22] Noriega R. E. R., Pinedo F. D. S., Arenas M. C., Urbina J. V., Figueroa H. E. H., Lakhtakia A. (2025). Opt. Eng..

[cit23] Zhang C., Zou C.-L., Zhao Y., Zhao Y., Dong C.-H., Wei C., Wang H., Liu Y., Guo G.-C., Yao J., Zhao Y. S. (2015). Sci. Adv..

[cit24] Kumar A. V., Rohullah M., Chosenyah M., Sindhuja G., Chandrasekar R. (2025). Angew. Chem., Int. Ed..

[cit25] Rohullah M., Chosenyah M., Kumar A. V., Chandrasekar R. (2025). Small.

[cit26] Liu H., Lu Z., Tang B., Qu C., Zhang Z., Zhang H. (2020). Angew. Chem., Int. Ed..

[cit27] Hayashi S., Yamamoto S.-y., Takeuchi D., Ie Y., Takagi K. (2018). Angew. Chem., Int. Ed..

[cit28] Takazawa K., Inoue J.-i., Mitsuishi K., Kuroda T. (2013). Adv. Funct. Mater..

[cit29] Hayashi S., Koizumi T. (2016). Angew. Chem., Int. Ed..

[cit30] Zhuo M.-P., Wu J.-J., Wang X.-D., Tao Y.-C., Yuan Y., Liao L.-S. (2019). Nat. Commun..

[cit31] Zhuo M.-P., Su Y., Qu Y.-K., Chen S., He G.-P., Yuan Y., Liu H., Tao Y.-C., Wang X.-D., Liao L.-S. (2021). Adv. Mater..

[cit32] Lv Q., Wang X.-D., Yu Y., Zhuo M.-P., Zheng M., Liao L.-S. (2022). Nat. Commun..

[cit33] Yin B., Gu J., Feng M., Zhang G. C., Zhang Z., Zhong J., Zhang C., Wen B., Zhao Y. S. (2019). Nanoscale.

[cit34] Wu B., Zhuo M.-P., Shi Y.-L., Gu L.-F., Zhao Y.-D., Su Y., Li Y.-Y., Lu H., Li W.-F., Wang Z.-S., Wang X.-D. (2025). Chem.

[cit35] Zhao S., Zhang J.-X., Wang L., Xu C.-F., Ma Y.-X., Wang X.-D., Liao L.-S. (2025). J. Am. Chem. Soc..

[cit36] Lan L., Li L., Qi J., Pan X., Di Q., Naumov P., Zhang H. (2023). Nat. Commun..

[cit37] Liu S., Lan L., Zhang H. (2025). Chem. Sci..

[cit38] Yang X., Lan L., Pan X., Di Q., Liu X., Li L., Naumov P., Zhang H. (2023). Nat. Commun..

[cit39] Liu K., Lei Y., Fu H. (2020). Chem. Mater..

[cit40] Lei Y., Sun Y., Zhang Y., Zhang H., Zhang H., Meng Z., Wong W.-Y., Yao J., Fu H. (2018). Nat. Commun..

[cit41] Feng C., Xu Z., Wang X., Yang H., Zheng L., Fu H. (2017). ACS Appl. Mater. Interfaces.

[cit42] Basak S., Chandrasekar R. (2010). Adv. Funct. Mater..

[cit43] Strohecker D. J., Lynch V. M., Holliday B. J., Jones R. A. (2017). Dalton Trans..

[cit44] Stanley J. M., Zhu X., Yang X., Holliday B. J. (2010). Inorg. Chem..

[cit45] Basak S., Narayana Y. S. L. V., Baumgarten M., Müllen K., Chandrasekar R. (2013). Macromolecules.

[cit46] Chandrasekhar N., Chandrasekar R. (2010). Chem. Commun..

[cit47] Narayana Y. S. L. V., Chandrasekar R. (2011). ChemPhysChem.

