# Supplementary material for: A chameleon-like core–shell organic/lanthanide flexible crystal waveguide for bandwidth and colour tunability
Source: Chem Sci. 2026 May 29;17(29):14178–84. doi: 10.1039/d6sc01977c (PMC13273597; doi:10.1039/d6sc01977c)
Supplement: SC-017-D6SC01977C-s001 [file SC-017-D6SC01977C-s001.pdf]

Supplementary Information (SI) for Chemical Science.
This journal is © The Royal Society of Chemistry 2026

# Supporting Information

## A Chameleon-like Core-Shell Organic/Lanthanide Flexible Crystal Waveguide for Bandwidth and Colour Tunability

*Melchi Chosenyah, Mehdi Rohullah, Avulu Vinod Kumar, K. V. Jovan Jose,* and Rajadurai Chandrasekar**

**Table of contents**


| Serial No. | Title | Page No. |
|---|---|---|
| 1 | Materials | 2 |
| 2 | a) Synthesis of BPP<br>b) Preparation of BPP microrods | 2 |
| 3 | Instrumental methods | 2-3 |
| 4 | Theoretical Raman spectral calculation | 4 |
| 5 | Figure S1: Optical images of un-coordinated and coordinated BPP | 4 |
| 6 | Figure S2: Optical and FESEM images of BPP with various lengths | 5 |
| 7 | Figure S3: Solid-state optical properties of the BPP and $Eu(tta)_3$ hydrate. | 5 |
| 8 | Figure S4: Optical waveguiding and FESEM micrographs of coordinated BPP microcrystal with EDX data | 6 |
| 9 | Figure S5: TEM images and SAED pattern of un-coordinated and coordinated BPP | 7 |
| 10 | Figure S6: Concentration dependent FL spectra of $Eu(tta)_3$ coordinated BPP | 7 |
| 11 | Figure S7: Micromanipulation of coordinated BPP crystal | 8 |
| 12 | Figure S8: Raman along with FL spectrum collected when excited the $Eu(tta)_3$ coordinated BPP with 532 nm | 8 |
| 13 | Table S1: CIE 1931 diagram's x and y coordinates | 9 |
| 14 | Figure S9: Two-point bending test of BPP on different substrate | 9 |
| 15 | References | 10 |

## 1. Materials

All chemicals and solvents like hexane, dichloromethane, toluene, and 1,4-dioxane were purchased from commercial sources (TCI chemicals, Sigma Aldrich, BLD chemicals, and Merck). Unless specified, HPLC solvents were used for synthesis and self-assembly. Europium(III) thenoyltrifluoroacetonate ($Eu(tta)_3$) hydrate, is purchased from Acros Organics.

## 2. Synthesis and preparation of BPP microcrystals

### a) Synthesis of BPP:[1]

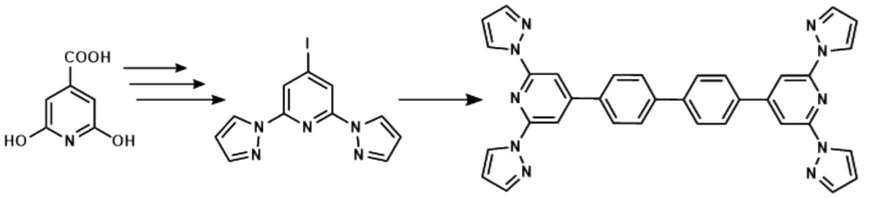


**Scheme S1** Synthetic scheme of BPP molecule. (Reaction conditions: 4,4'-biphenylenediboronicacid, $[Pd(PPh_3)_4]$ and 2M $Na_2CO_3$/1,4-Dioxane/72 hours/70 °C).[1]

### b) Preparation of BPP microrods

For self-assembly studies, in a clean vial, BPP (0.2 mg) was dissolved in DCM (1 mL) and left undisturbed at rt for 6 h. Drop casting 2-3 drops of this DCM solution of BPP onto a clean glass coverslip under hexane atmosphere produced microrods after complete evaporation of the solvent.[2]

## 3. Instrumental methods

a) **Solid-state absorption and emission studies:** The solid-state absorption spectra were recorded using a Shimadzu UV-3600 spectrometer operating in diffuse reflectance UV–visible (DR–UV–vis) mode. The obtained reflectance data were converted into absorbance spectra using the Kubelka–Munk function. Solid-state emission measurements were carried out on a JASCO FP-8500 spectrofluorometer.

b) **Confocal optical microspectroscopy studies:** The optical waveguiding experiments of a single uncoordinated and Eu-coordinated BPP microcrystals were carried out on a backscattering mode setup of the Wi-Tec alpha 300 AR laser confocal optical microscope (LCOM) equipped with a Peltier-cooled CCD detector. The spectra were recorded using the following parameters: 300 grooves/mm grating BLZ = 750 nm, the accumulation time = 10, and the integration time = 0.5 s per spectra. 355/405 nm, 532 nm, and 785 nm lasers were used as excitation sources. The excitation of the sample was performed using 60× objective. The output signal was collected using 20× (N.A. = 0.6) objective. The signal was sent to a CCD detector through a multimode optical fiber of

diameter 100 µm (3 µm core). All measurements were performed at ambient condition and images were processed using WI-TEC software. The spectra were baseline corrected.

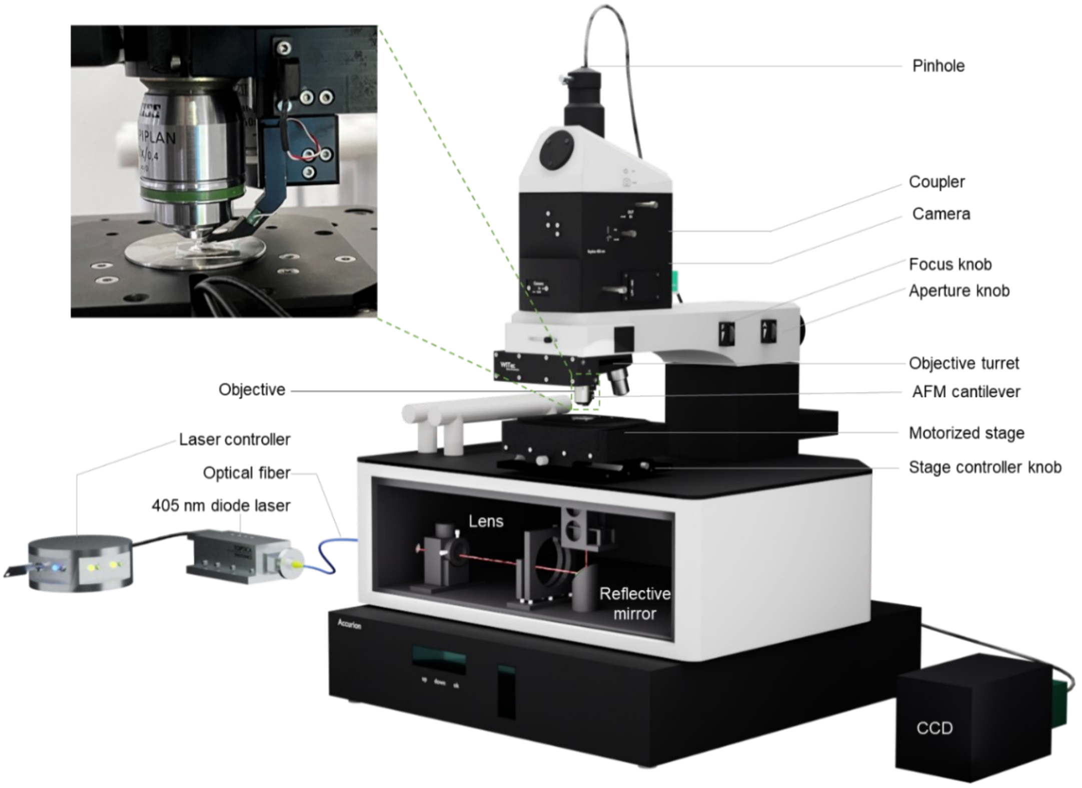


**Scheme S2** Graphical illustration of confocal optical microscopy. Inset shows the image of attached AFM cantilever tip for the crystal manipulation.

c) **Mechanical micromanipulation of the BPP microcrystals:** The BPP microcrystals were mechanically micromanipulated using an AFM cantilever tip (Adama: NM-TC, with a force constant of 350 N/m and a typical tip radius of 25 +/- 10 nm) attached to the above-mentioned WiTec confocal optical microscope setup. The optical microscope was used to visualize the microcrystals.

d) **Field emission scanning electron (FESEM) microscopy studies:** The energy dispersive X-ray (EDX) analysis and morphological analysis of the BPP microrods were performed using a Zeiss FESEM operating at 3 kV. Samples were coated with thin layer of gold before imaging.

e) **Transmission electron microscopy (TEM) studies:** The microcrystals of BPP drop casted onto a carbon coated copper grids were probed for their morphology and crystallinity using a (JEOL F200) TEM, 200 kV accelerating voltage. CrystBox software was used to analyse selected area electron diffraction (SAED) pattern.

## 4. Theoretical Raman spectral calculations

The molecular geometry optimization is performed on the ligand (BPP), Eu(tta)$_3$ hydrate, and Eu(tta)$_3$ coordinated BPP complexes at Becke three parameters hybrid functional with Lee–Yang–Parr correlation functional (B3LYP) level of theory with Def2svp basis set, using the Gaussian09 suite of programs.[3] The local minimal characteristics of the optimized geometries of Eu(tta)$_3$ coordinated BPP complex is confirmed by the absence of imaginary normal vibrational modes and optimized structure is depicted in Figure 3a. The Raman spectra of the ligand and coordination complexes are calculated at the same level of theory and basis set on the optimized geometries. Figure 3c depicts the Raman spectra of Eu(tta)$_3$ coordinated BPP complex and a comparison with experimental spectra is presented in the results and discussion session.

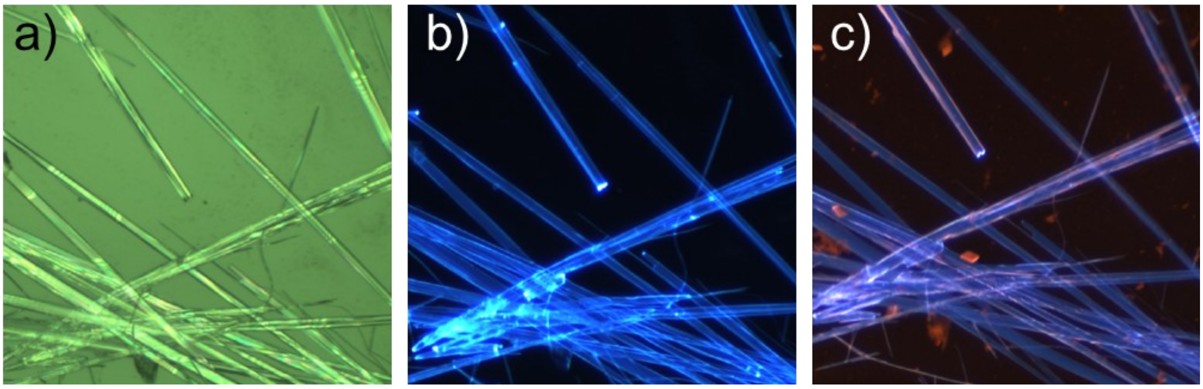


**Figure S1** a) Confocal optical image of BPP microcrystals. FL images of b) Un-coordinated and c) Coordinated BPP microcrystals.

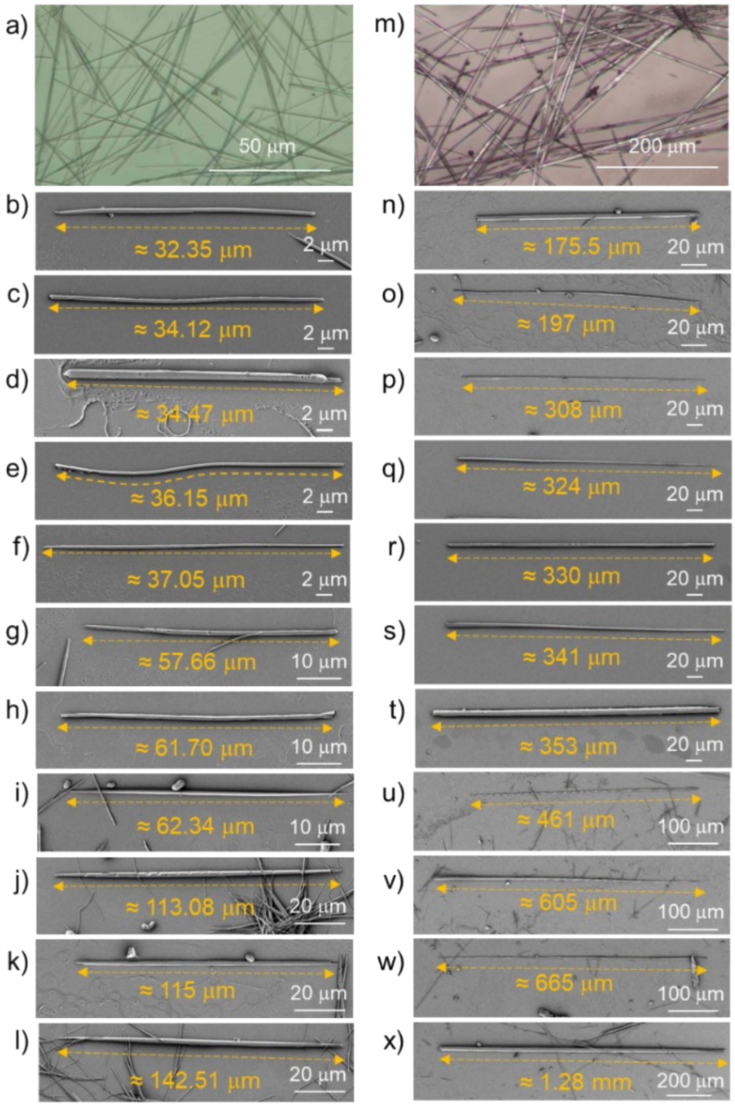


**Figure S2** a) Optical and b-l) FESEM images of the BPP microcrystal with the varying length from ≈ 32 μm to ≈ 142 μm. m) Optical and n-x) FESEM images of the BPP microcrystal with the varying length from ≈ 175 μm to ≈ 1.28 mm.

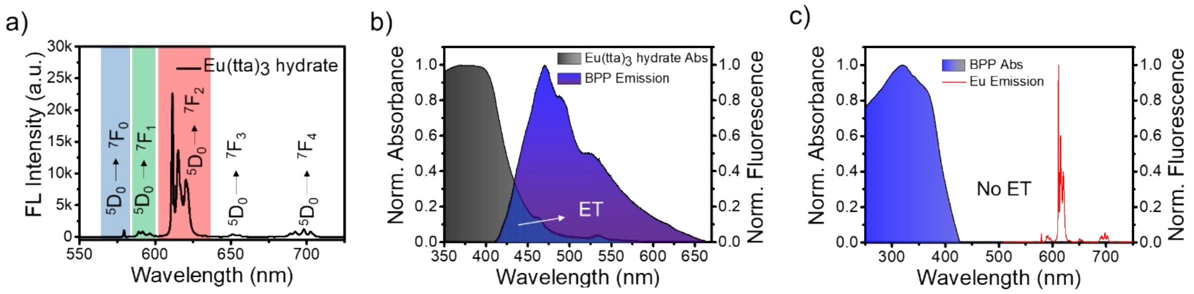


**Figure S3** Solid-state optical properties of BPP and Eu(tta)$_3$ hydrate. a) Hypersensitive narrow band *f-f* transitions of Eu(III) ion. b) Overlap of absorption and emission spectra of Eu(tta)$_3$ hydrate and BPP, respectively demonstrating the radiative energy transfer from BPP to Eu(III) ion. c) The absorption and emission spectra of BPP and Eu(tta)$_3$ hydrate.

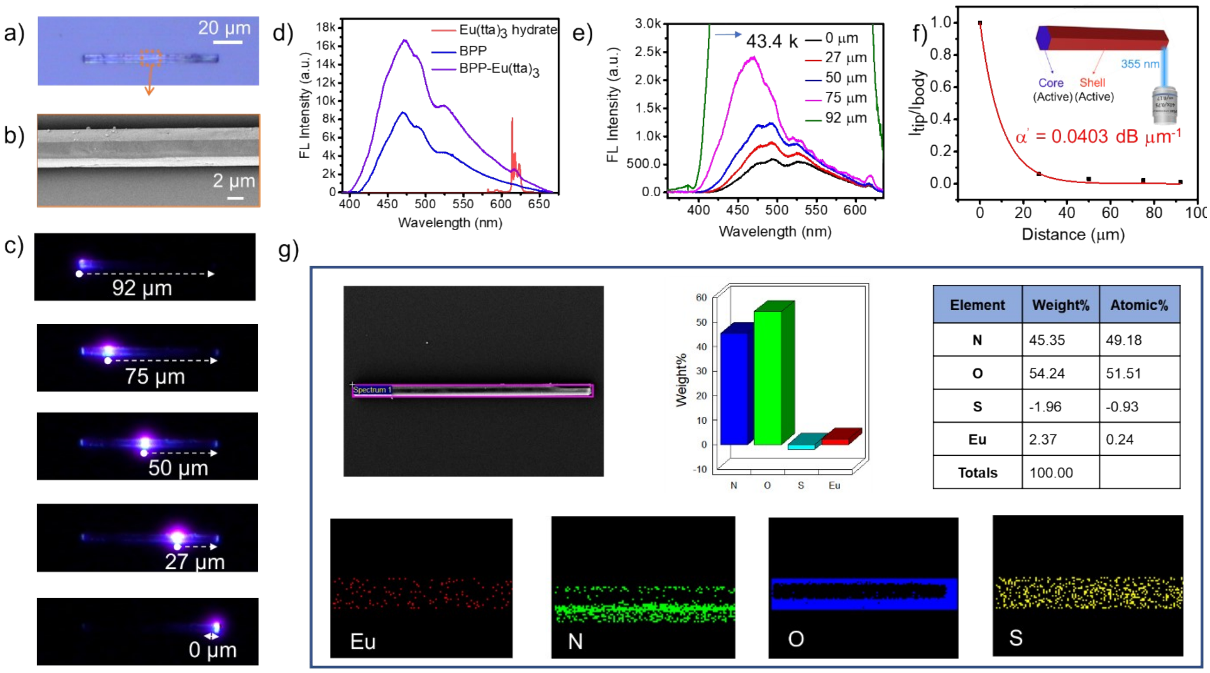


**Figure S4** a) Confocal optical image, b) FESEM image, and c) FL images of Eu(tta)$_3$ coordinated BPP microcrystal active waveguide excited at different positions. d) Spectral comparison of BPP waveguide, Eu(tta)$_3$ hydrate, and BPP-Eu(tta)$_3$ crystal waveguide. e) The excitation position-dependent FL spectra obtained from the BPP-Eu(tta)$_3$ crystal waveguide shown in Figure S4c. f) The plot used for the estimation of optical loss of BPP-Eu(tta)$_3$ crystal waveguide. Inset: Graphical representation of the optical waveguiding experiment. g) FESEM-EDX data collected in a selected area on BPP-Eu(tta)$_3$ crystal waveguide confirming the presence of Eu element.

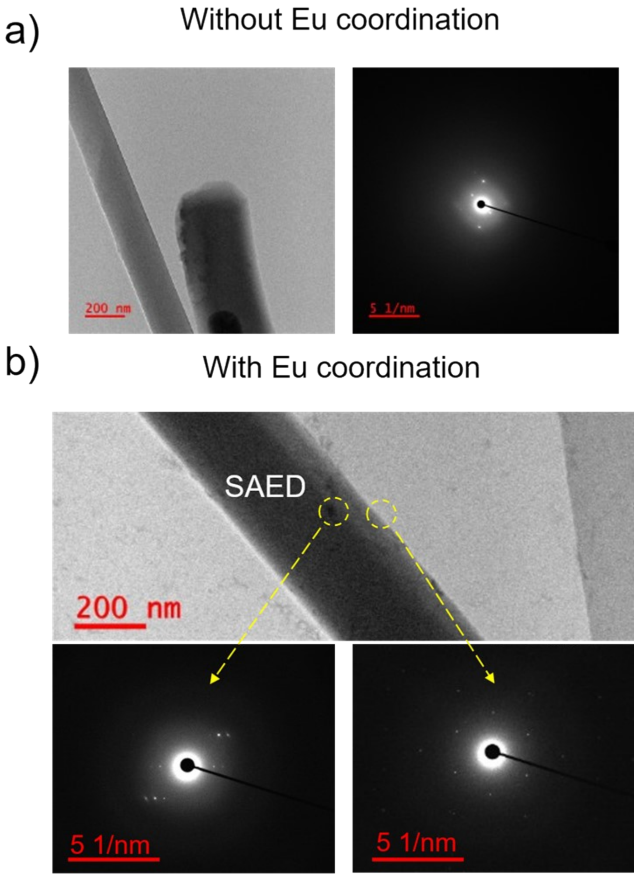


**Figure S5** TEM images and selected area diffraction pattern(s) of a) un-coordinated and b) coordinated BPP microcrystals.

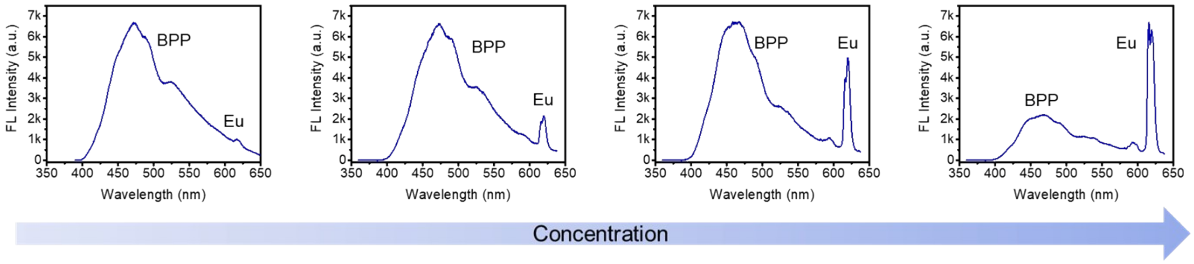


**Figure S6** FL spectra of $Eu(tta)_3$ coordinated BPP with varying Eu concentration.

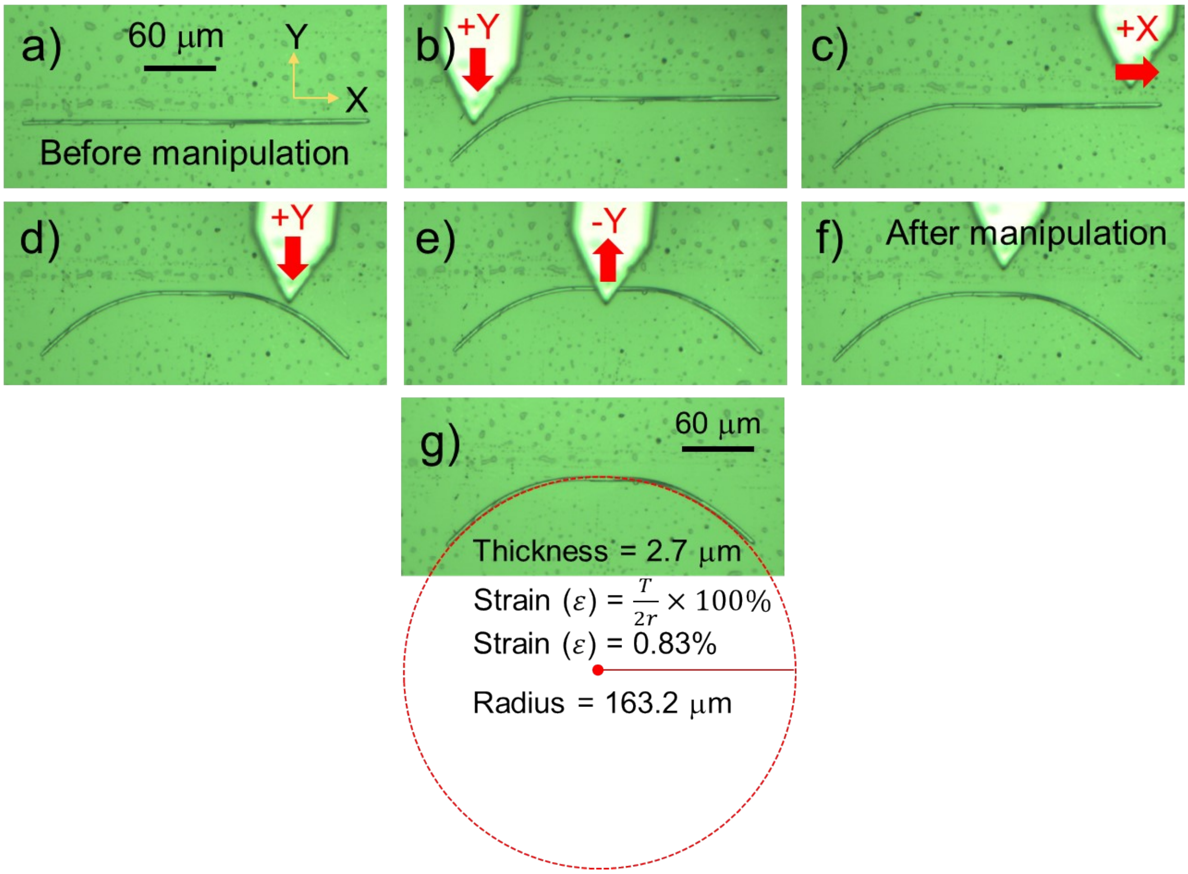


**Figure S7** a-f) Mechanical micromanipulation of Eu coordinated BPP crystal using AFM cantilever tip. g) The strain value estimated from the bent crystal.

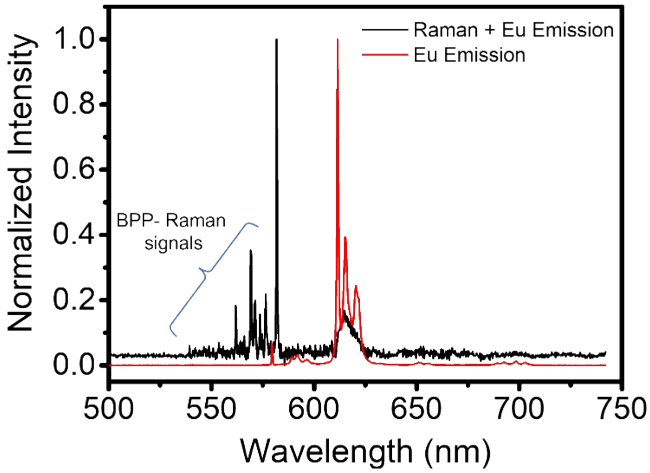


**Figure S8** Raman spectrum of BPP along with FL spectrum of Eu(III) recorded from Eu(tta)$_3$ coordinated to BPP exciting with a 532 nm laser. Note: The 532 nm light falls outside the optical absorption window of BPP, whereas it falls within the optical absorption window of Eu(III). For comparison the FL spectrum of Eu(tta)$_3$ ($\lambda_{ex}$ = 532 nm) is overlapped with the spectrum of Eu(tta)$_3$ coordinated to BPP.

**Table S1** The CIE 1931 diagram's x, y coordinates (colour coordinates) for BPP, $Eu(tta)_3$ hydrate, and Eu coordinated BPP when irradiated with 355 nm, 532 nm and 785 nm Lasers.

| Emission source | CIE 1931 | |
|---|---|---|
| | x | y |
| BPP | 0.20261 | 0.28014 |
| $Eu(tta)_3$ hydrate | 0.66668 | 0.32127 |
| Eu coordinated BPP ($\lambda_{ex}$ = 355 nm) | 0.21097 | 0.2808 |
| Eu coordinated BPP ($\lambda_{ex}$ = 532 nm) | 0.33627 | 0.39639 |
| Eu coordinated BPP ($\lambda_{ex}$ = 785 nm) | 0.73234 | 0.26766 |

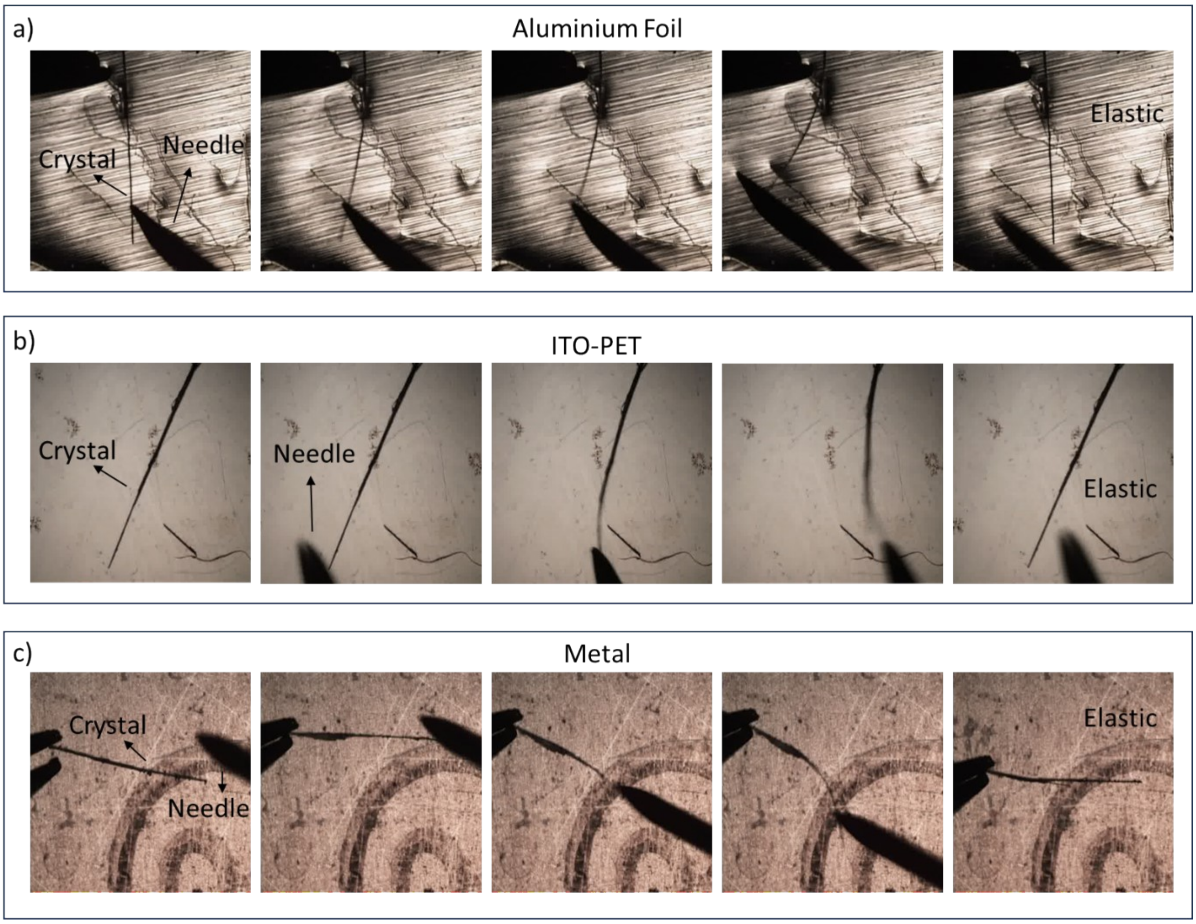


**Figure S9** a-c) Optical images depicting the two-point bending test performed on BPP crystal using tweezers and needle to demonstrate crystal flexibility on aluminium, ITO–PET, and Cu metallic surfaces.

## 5. References


[1] N. Chandrasekhar, R. Chandrasekar, *Chem. Commun.,* 2010, **46**, 2915.

[2] A. V. Kumar, M. Rohullah, M. Chosenyah, J. Ravi, U. Venkataramudu, R. Chandrasekar, *Angew. Chem. Int. Ed.,* 2023, **62**, e202300046.

[3] Gaussian 09, Revision C.01, M. J. Frisch, G. W. Trucks, H. B. Schlegel, G. E. Scuseria, M.A. Robb, J. R. Cheeseman, G. Scalmani, V. Barone, B. Mennucci, G. A. Petersson, H.Nakatsuji, M. Caricato, X. Li, H. P. Hratchian, A. F. Izmaylov, J. Bloino, G. Zheng, J. L.Sonnenberg, M. Hada, M. Ehara, K. Toyota, R. Fukuda, J. Hasegawa, M. Ishida, T. Nakajima,Y. Honda, O. Kitao, H. Nakai, T. Vreven, J. A. Montgomery, Jr., J. E. Peralta, F. Ogliaro, M.Bearpark, J. J. Heyd, E. Brothers, K. N. Kudin, V. N. Staroverov, R. Kobayashi, J. Normand,K. Raghavachari, A. Rendell, J. C. Burant, S. S. Iyengar, J. Tomasi, M. Cossi, N. Rega, J. M.Millam, M. Klene, J. E. Knox, J. B. Cross, V. Bakken, C. Adamo, J. Jaramillo, R. Gomperts, R.E. Stratmann, O. Yazyev, A. J. Austin, R. Cammi, C. Pomelli, J. W. Ochterski, R. L. Martin, K.Morokuma, V. G. Zakrzewski, G. A. Voth, P. Salvador, J. J. Dannenberg, S. Dapprich, A. D.Daniels, Ö. Farkas, J. B. Foresman, J. V. Ortiz, J. Cioslowski, and D. J. Fox, Gaussian, Inc.,Wallingford CT, 2009.
